# The role of schools as an opportunity for transmission of local knowledge about useful Restinga plants: experiences in southeastern Brazil

**DOI:** 10.1186/s13002-021-00461-0

**Published:** 2021-05-17

**Authors:** Nicky van Luijk, Gustavo Taboada Soldati, Viviane Stern da Fonseca-Kruel

**Affiliations:** 1 Programa de Pós-Graduação em Biodiversidade em Unidades de Conservação, Escola Nacional de Botânica Tropical - Instituto de Pesquisas Jardim Botânico do Rio de Janeiro, Rua Pacheco Leão, 2040, Jardim Botânico, Rio de Janeiro, Brazil; 2 Departamento de Botânica, Universidade Federal de Juiz de Fora, Rua José Lourenço Kelmer, S/N, São Pedro, Minas Gerais Juiz de Fora, Brazil; 3 Diretoria de Pesquisas, Instituto de Pesquisas Jardim Botânico do Rio de Janeiro, Rua Pacheco Leão, 915, Jardim Botânico, Rio de Janeiro, Brazil

**Keywords:** Social learning, Biocultural traits, Local ecological knowledge, Adolescence, Cultural evolution, Adaptive memory

## Abstract

**Background:**

The study of cultural transmission can help identify processes that influence knowledge systems dynamics and evolution, especially during childhood and youth, which are fundamental phases in acquiring survival skills. In this sense, we use the knowledge about useful restinga plants (Brazilian coastal vegetation) as an analytical model to describe, compare, and analyze cultural transmission during youth, while factoring in origin, in the Cabo Frio region, southeastern Brazil. We tested (1) whether transmission of knowledge is conservative, (2) whether immigration events define the transmission modes, (3) whether teaching is the most important social transmission cognitive process, and (4) which type of stimulus/context is most important for the knowledge transmission process.

**Methods:**

Questionnaires and free listings were applied to 150 high school students aged between 15 and 20 to obtain information about socioeconomic characteristics, useful plant knowledge, and cultural transmission. We analyzed the distribution of knowledge according to the informant’s origin and evaluated the models, processes, and context with which this information was transmitted. The chi-square test was used to determine the association between origin, plant knowledge, and transmission as well as to reveal the most important models, modes, and processes during youth.

**Results:**

Informants provided 299 plant citations ($\overline{x}$ = 1.75; s = 1.73) related to 37 species. The categories of the most cited uses were edible (93) and medicinal (32). Statistical results showed that origin did not influence knowledge distribution and transmission. In addition, although the most relevant mode was the conservative (vertical) one, the one-to-many diffuse mode (teacher) was highlighted. The new environmental context for immigrants did not influence transmission, the main transmission process was teaching, and the learning contexts were predominantly school-related.

**Conclusion:**

Plant knowledge in youth was related to local edible and medicinal plants, indicating adaptive knowledge linked to material demands for survival. While the initial models for cultural transmission are family (vertical), during the development phase of juveniles, other actors become models (one-to-many). In addition, the nature of the information (survival demand) and age are more relevant to cultural transmission than the socio-environmental context.

**Supplementary Information:**

The online version contains supplementary material available at 10.1186/s13002-021-00461-0.

## Background

Humans are a social species that possess a set of accumulated and shared cultural information systems that are not static, but are subject to change [1, 2]. The cultural evolution theory presents theoretical foundations to explain the spatiotemporal development of these systems using Darwinian conceptions of evolution [2]. According to this theory, change in the frequencies of information is defined by micro-evolutionary processes such as cultural transmission [2]. Hewllet and Cavalli-Sforza [1] proposed that the cultural transmission can be classified according to the learning models, with different implications for information propagation speed, in four modes: vertical (parents to children, slow in the information dissemination, therefore conservative), horizontal (same generation pairs, fast in the dissemination, therefore diffusive), oblique many-to-one mode (different generations pairs, conservative), and oblique one-to-many (one individual transmitting information to several generations, diffusive). The way cultural information is shared between people affects the structure and evolution of the knowledge system because it enables cultural information to be fixed, altered, or lost [2]. In addition, knowledge is acquired by different cognitive processes, such as observation, imitation, and teaching [3]. Among these processes, teaching is the one that most favors the reliable transmission of information, since the model is intended to guarantee the apprentice’s learning [3]. The learning models and cognitive process are independent and not mutually exclusive, for example, a child can learn from his parents (vertical mode) through the processes of teaching, or observation, or imitation [3]. But, the only process that promotes cultural evolution is teaching, because it allows increases adaptability through effective information sharing [4].

From this evolutionary perspective, some cultural information, like plant knowledge (see [5]), undergoes strong selective pressure because it is linked to survival, and it is therefore promising in the understanding of cultural evolution [4]. Investigations suggest that childhood is the most important age for learning, with parents being the main models for the transmission of knowledge (vertical mode), which occurs mainly in practical and everyday activities [1, 5–11]. These same studies indicate that learning continues in adolescence, when other transmission processes and models are used. Fundamental knowledge about survival, such as knowledge regarding information about medicinal plants [12], food plants [5], and hunting strategies [9], are learned during childhood, sustaining the thesis of an adaptive memory [13] and the human naturalistic mind [14]. Knowledge is not transmitted randomly. Rather, it depends on selection processes such as the model’s prestige [15, 16] and knowledge’s inherent characteristics such as reliability and safety [5]. These studies indicate microprocesses that drive cultural adaptations and define the distribution and structure of local knowledge from an evolutionary perspective. In addition to the transmission of knowledge, it is important to note that local knowledge is also influenced by socioeconomic factors such as age, gender, ethnicity, and origin, which are occasionally reflected in different distribution patterns of knowledge domains between community members [17–19]. 

Although there is a consensus that culture is dynamic and can adapt to new ways of living, some factors related to globalization and urbanization seem to be forces that generate transformations in knowledge, such as immigration, formal schooling, and the technology use [20–22]. Studies have shown that international immigration leads to the information incorporation from the new environment or that the foreigners adapt the one they bring from their place of origin [22]. However, migrations on a regional scale present different results, showing that in peri-urban communities the place of birth may not influence the acquisition of knowledge about plants [23], or may favor the incorporation of local knowledge by immigrants [24]. Regarding formal schooling, there is evidence that it reflects negatively on traditional knowledge when it is not contextualized [25–28]. Moreover, the new resources provided by modernization, such as schooling and new means of communication and information technology (internet, television, books) can provide new opportunities for social learning [20–26] (a multistage process [29]), adding new dimensions to cultural transmission that are not yet well understood and can be explored in ethnobotanical research.

The modernization provided by industrialization has not only affected cultural systems but also threatened global biodiversity since the beginning of the Anthropocene [30]. Regions of high plant richness are weakened, as is the case of restinga, a coastal environment associated with the tropical complex of the Brazilian Atlantic forest [31].

The restinga is heterogenic in different aspects, such as environmental, floristic, and related to different human groups (fishermen, quilombolas or maroon communities, rural communities) that occupy its adjacent areas and are under pressure [32]. The degradation and suppression of restinga vegetation dates back to five centuries and future projections for the preservation of its remnants are not positive [33]. The impacts currently come from real estate speculation, accelerated disordered urbanization, ornamental plants extraction, and exotic species introduction [31, 33]. Given the importance of this ecosystem for Brazilian biodiversity and for the survival and identity of Brazilian traditional communities [32], the understanding of cultural transmission of knowledge related to restinga plants can have positive impacts on species conservation and management, as well as on the biocultural conservation and comprehension of the local knowledge system dynamics.

In this context, the contribution of this work is to understand the maintenance and development of the Brazilian biocultural heritage regarding to immigration in a plant diversity hotspot region (Cabo Frio Center of Plant Diversity) [34], in a school context, based on the assumption that the environment (restinga) influences people’s knowledge. We seek to contribute with a case study comparing knowledge about restinga plants and describing the models, processes, and contexts of cultural transmission among native and non-native (immigrants) school students (juveniles) in recently urbanized area of southeastern Brazil, with the presence of traditional communities. We hypothesized that the two groups would present differences in the knowledge held about restinga and in the way of acquiring it. This is because, for the local communities, this vegetation was the main source of available resources until the offer of modern facilities increased by the arrival of industrialization in the 1950s. Industrialization also generated great immigration and vegetation suppression, influencing the livelihoods and the knowledge dynamics.

We used knowledge about useful restinga plants as an analytical model, assuming that this cultural information is linked to survival material demands important in the sociocultural context of the traditional communities, and would therefore provide valuable information about cultural evolution. Assuming that all knowledge is constructed and linked with a specific context [5], we worked in a school context within an area with a historical presence of artisanal fisherman communities, which has received a significant immigration flow in the last decades. We used the origin of the students and the characteristics of the information transmitted, such as the use and geographical distribution of the species, to reflect on cultural evolution. We tested (1) whether the transmission of knowledge is conservative (H1), based on the premise that vertical transmission is the most frequent mode used; (2) whether immigration events define the transmission modes, considering the premise that non-natives would learn mainly through horizontal transmission (that is, with native colleagues) (H2); (3) whether teaching is the most important social transmission cognitive process (see [4]) (H3); and, finally, (4) which type of stimulus/context is most important for the knowledge transmission process (H4).

## Methods

### Study area

**Fig. 1:**
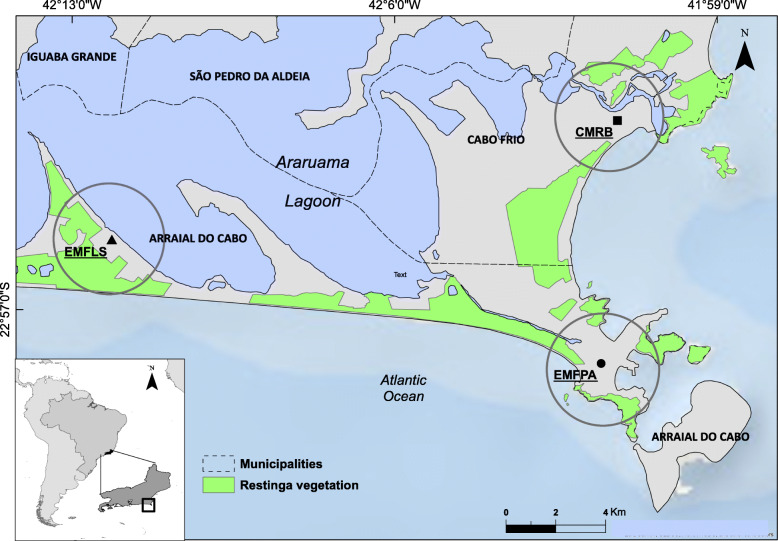
Study area and EMFPA, EMFLS, and CMRB schools, Rio de Janeiro state, Brazil (CSCG/JBRJ)

This study was conducted in the “Cabo Frio Region,” in the municipalities of Arraial do Cabo (22° 57′ 58″ and 42° 01′ 40″), and Cabo Frio (22° 52′ 46″ and 42° 01′ 07″), on the southeastern coast of Brazil (Fig. 1). In this region (sandy coastal plains of Quaternary origin associated with the Atlantic Forest biome), coastal vegetation called *restinga*, which has high diversity and endemism in a mosaic of plant communities, predominates [31, 35]. This region presents climatic peculiarities with a rainfall index below 900 mm per year, 24 °C average annual temperature, and strong winds from the northeast quadrant [35].

The history of human occupation by communities of prehistoric hunters and gatherers in the Cabo Frio region dates back at least 6000 years [36]. It remained occupied by Tupinambás indigenous communities until the sixteenth century, when great changes began with the period of Portuguese colonization, African slave trade, and consequent ethnic miscegenation [37]. However, the human population of Arraial do Cabo remained in isolation longer than the population in Cabo Frio due to its geographical situation (it was a peninsula), and the people lived in small villages on marine and restinga resources [38]. However, local livelihoods changed dramatically from 1950 due to the intensification of the salt industry, urbanization, immigration, and tourism, which affected plant and cultural diversity [38]. Currently, Arraial do Cabo has approximately 27,715 inhabitants and it is 158,952 km^2^ in size, while Cabo Frio has 186,227 inhabitants and it is 410,418 km^2^ in size [39, 40]. In the last 10 years, the region has shown a demographic growth of 41% [39–41] due to immigration from the capital of the state of Rio de Janeiro and the northeast coast of Brazil, reinforced by real estate speculation. The main economic activities are summer tourism (service sector) and local commerce.

### Data collection and analysis

Fieldwork was conducted from March to December 2017 in three public schools: Francisco Porto Aguiar Municipal School (EMFPA) and Francisco Luiz Sobrinho Municipal School (EMFLS) in Arraial do Cabo, and Rui Barbosa Municipal School (CMRB) in Cabo Frio (Fig. 1). The selection of schools was based on the proximity of the main schools in Cabo Frio region to the remaining areas of native restinga vegetation, excluding schools over a distance of more than 1.5 km from this natural environment. All three schools selected are 1.2 km away from restinga areas. This approach was used because in the study region there are other types of vegetation besides restinga, such as highland forests. A total of 150 youth, of whom 70 were natives and 80 were non-natives, participated in the interviews (92 females and 58 males). Individual questionnaires were applied in the classroom to students aged between 15 and 20 years from eight classes. The questionnaire had three categories of questions: (1) socioeconomic data (age, gender, place of birth, parents’ natural state, and student and family’s place of residence), (2) local knowledge about restinga plants and their uses (the free listing technique was used for this) [42]; and (3) how, when, and with whom they learned about restinga plants (to infer about cultural transmission) [5]. The first category enabled us to draw a social profile of the informants, required to better understand the distribution of local knowledge related to (H1) and (H3). These data were also necessary to test (H2), namely, that young migrants learn more through diffusive modes. The second category was used to characterize local knowledge and, thus, obtain a more comprehensive understanding of the information transfer processes. The third category aimed to identify the processes, contexts, and models of social learning, information necessary to test all four hypotheses established here.

Students were categorized as natives (young people who were born in the municipality of study and who have at least one native parent) and non-natives (young people who were not born in the municipality of study and/or have immigrant parents). Natives of the Cabo Frio region are European, African, and indigenous descents and are not from a single ethnic group. Therefore, natives and non-natives speak the same language (Portuguese). Such an approach was adopted because of the great immigration that occurred in the study region, which could have possibly reflected in native flora knowledge distributions and transmission processes differing between natives and non-natives, corresponding to (H2). This study is part of major investigations conducted by Rio de Janeiro Botanical Garden Research Institute on local restinga flora, involving botanical, floristic, ethnobotanical, and conservationist aspects. Botanical material was collected and taxonomically identified by this research group and deposited in Herbariums of the Federal Rural University of Rio de Janeiro (RBR) and the Rio de Janeiro Botanical Garden Research Institute (RB). Therefore, vernacular names (which are general for the area) and species identification were made in previous ethnobotanical studies and used to recognize and compare information [43, 44], corroborating the vernacular names mentioned in the interviews by visual stimuli [45], showing photographs to the informants, without associated data, after completing the questionnaires to verify knowledge.

The free listings were analyzed by Smith’s Salience Index using the software ATHROPAC 4.98. Plant species mentioned in the interviews were classified as native or exotic restinga vegetation using the Brazil Flora G [46]. First, the general knowledge obtained in the interviews was evaluated and then the domain of knowledge about native restinga plants was analyzed. The information considered for inferential statistical analysis and conservational approach was only about the native species that grow in the Cabo Frio Region [47]. The plant uses were analyzed and categorized as edible, medicinal, and ornamental, and used in dyeing, construction, cosmetics, and handicrafts.

To characterize the models of cultural transmission (H1 and H2), we considered “vertical” (parents or grandparents), “horizontal” (friends of the same age group), “oblique many-to-one” (a family member other than parents and grandparents, or older people with no kinship), and “oblique one-to-many” (someone of prestige, like a teacher, nutritionist, scoutmaster, or the media) [1]. Information through technology (internet, television, books, and newspapers) was considered “media” [48]. In order to understand the transmission processes (H3), the responses were categorized into “teaching” (processes in which a model made a conscious effort to facilitate the learning of the young, with no differentiation between formal and informal education) or “observation” (transmission conditioned by the simple attention of the young to the action of a model) [3]. The context or stimulus (H4) that triggered the learning process was categorized as “school classes” (when learning occurred in activities at school), “conversations” (when learning occurred in moments of dialog), “visiting the restinga environment” (when learning occurred during nature visits with family, friends, or a prestigious person), “family interaction” (when learning occurred through the family), “accessing media” (when learning occurred through the use of the internet, television, books, and/or newspapers), and “observation of medical and food preparation” (when learning occurred by observing someone manipulating plant resources).

To test the first hypothesis, namely, that conservative (vertical) pathways are more important among students, we analyzed the adherence of frequency of citations of the transmission categories (4×1 matrix) to the matrix of equal expected proportions (4×1), using the chi-square test. If the null hypothesis was rejected, an analysis of adherence was performed considering all combinations of the learning categories. The chi-square test was also used to analyze the second hypothesis, namely, the interdependence between geographic origin and transmission routes (2×4 matrix). This test also compared the frequency adherence of the transmission mechanisms (3×1 matrix) with a matrix of equal expected proportions (3×1). Finally, the distribution of frequencies in the categories of learning stimuli was compared with a distribution of equal proportions using the chi-square test. All analyses were conducted using Program R, version 4.02 [49].

## Results

### Which plants do the youth know?

The young participants provided 299 plant citations (mean $\overline{x}$ = 1.75; standard deviation s = 1.73) from 37 species, belonging to 23 families (Table 1). They cited between 1 and 8 species of plants (with a median of 2), but 31% of the students did not mention any plants (*N* = 47). The most cited species were *Pilosocereus arrabidae* (76), *Allagoptera arenaria* (36), *Eugenia uniflora* (33), *Chrysobalanus icaco* (22), *Schinus terebinthifolia* (15), and *Anacardium occidentale* (12). Therefore, the families best represented were Arecaceae (4), Cactaceae (4), Anacardiaceae (3), and Myrtaceae (3). Only one or two species from other families were cited.

**Table 1:** Plants and uses mentioned by the students of the schools EMFPA, EMFLS, and CMRB

Family	Specie (taxon)	Local name	Origin	Frequency of citation	Salience index	Categories of uses
Cactaceae	*Pilosocereus arrabidae* (Lem.) Byles & Rowley	Cacto	Native	76 (n39, nn37)	0.580	3 (e18, o6, d2)
Arecaceae	*Allagoptera arenaria* (Gomes) Kuntze	Guriri	Native	36 (n21, nn15)	0.253	1 (e19)
Myrtaceae	*Eugenia uniflora* L.	Pitanga	Native	33 (n17, nn16)	0.205	2 (e20, m2)
Chrysobalanaceae	*Chrysobalanus icaco* L.	Bajirú	Native	22 (n4, nn18)	0.141	2 (e3, m6)
Anacardiaceae	*Schinus terebinthifolia* (Mart.) Engl.	Aroeira	Native	15 (n6, nn9)	0.105	2 (e5, m11)
Anacardiaceae	*Anacardium occidentale* L.	Caju	Native	12 (n6, nn6)	0.069	1 (e10)
Bromeliaceae	*Neoregelia cruenta* (R.Graham) L.B.Sm.	Bromélia	Native	9 (n4, nn5)	0.047	1 (o2)
Sapotaceae	*Pouteria caimito* (Ruiz & Pav.) Radlk	Guapeba	Native	8 (n4, nn4)	0.037	2 (e4, c1)
Orchidaceae	*Cattleya guttata* Lindl.	Orquídea	Native	8 (n7, nn1)	0.049	1 (o4)
Casuarinaceae	*Casuarina equisetifolia* L.	Casuarina	Exotic	8 (n5, nn3)	0.048	No uses cited
Blechnaceae	*Telmatoblechnum serrulatum* (Rich.) Perrie, D.J. Ohlsen & Brownsey	Samambaia	Native	7 (n4, 3nn)	0.038	No uses cited
Lamiaceae	*Plectranthus barbatus* Andr.	Boldo	Exotic	9 (n5, nn4)	0.043	2 (e1, m5)
Amaranthaceae	*Dysphania ambrosioides* (L.) Mosyakin & Clemants	Erva-de-santa-maria	Native	6 (n2, nn4)	0.023	1 (m5)
Myrtaceae	*Myrciaria floribunda* (H. West ex Willd.) O. Berg.	Cambium	Native	6 (n5, nn1)	0.034	1 (e4)
Fabaceae	*Mimosa pudica* L.	Dormideira	Native	6 (n5, nn1)	0.040	No uses cited
Asparagaceae	*Aloe vera* L.	Babosa	Exotic	4 (n1, nn3)	0.033	1 (cos4)
Arecaceae	*Cocos nucifera* L.	Coqueiro	Exotic	4 (n2, nn2)	0.021	1 (e1)
Malpighiaceae	*Malpighia emarginata* D.C.	Acerola	Exotic	3 (n2, nn1)	0.016	1 (e3)
Clusiaceae	*Clusia fluminensis* Planch. & Triana	Clusia	Native	3 (n1, nn2)	0.019	No uses cited
Convolvulaceae	*Ipomoea pes-caprae* (L.) R.Br.	Salsa-da-praia	Native	2 (n2, nn0)	0.011	No uses cited
Arecaceae	*Bactris setosa* Mart.	Tucum	Native	2 (n1, nn1)	0.010	No uses cited
Arecaceae	*Philodendron corcovadense* Kunth	Cipó-imbê	Native	2 (n1, nn1)	0.009	1 (h1)
Urticaceae	Indeterminate	Ortiga	Exotic	2 (n1, nn1)	0.011	No uses cited
Asteraceae	*Taraxacum officinale* (L.) Weber ex F.H.Wigg	Dente-de-leão	Exotic	2 (n1, nn1)	0.006	1 (o1)
Clusiaceae	*Garcinia brasiliensis* Mart.	Bacupari	Native	1 (n0, nn1)	0.004	2 (e1, m2)
Cactaceae	*Melocactus violaceus* Pfeiff.	Coroa-de-frade	Native	2 (n2, nn0)	0.008	No uses cited
Turneraceae	*Turnera ulmifolia* L.	Maria-vai-com-outras	Exotic	1 (n1, nn0)	0.014	1 (o1)
Bignoniaceae	*Handroanthus* sp.	Ipê	Native	1 (n1, nn0)	0.003	No uses cited
Cactaceae	*Brasiliopuntia* brasiliensis (Willd.) A.Berger	Arumbeba	Native	1 (n1, nn0)	0.002	1 (e1)
Lamiaceae	*Melissa officinalis* L.	Erva-cidreira	Exotic	1 (n0, nn1)	0.003	1 (m1)
Myrtaceae	*Eugenia selloi* (O. Berg) B.D. Jacks.	Pitangobaia	Native	1 (n0, nn1)	0.008	1 (e1)
Anacardiaceae	*Spondias purpurea* L.	Siriguela	Exotic	1 (n0, nn1)	0.003	No uses cited
Typhaceae	*Typha domingensis* Pers.	Tifa	Native	1 (n1, nn0)	0.010	No uses cited
Cactaceae	*Cereus fernambucensis* Lem	Cacto-caldo	Native	1 (n0, nn1)	0.010	1 (e1)
Asteraceae	*Helianthus annuus* L*.*	Girassol	Exotic	1 (n0, nn1)	0.006	No uses cited
Sapotaceae	*Mimusops coriacea* (A. DC.) Miq.	Abricó	Exotic	1 (n0, nn1)	0.006	No uses cited
Rutaceae	*Citrus x limon* (L.) Osbeck	Limão	Exotic	1 (n1, nn0)	0.002	1 (e1)

In order of specie frequency of citation. Botanical family, scientific name, local name, geographic distribution. General total of plant citations (frequency of citation by n—natives, nn—nonnatives). Total use categories (frequency of citation of uses per use category: e—edible, o—ornamental, m—medicinal, d—dyeing, c—construction, cos—cosmetic, h—handicraft)

The plant citation frequencies and species richness according to the origin of the youth (see Additional file: Table 2) revealed that plant knowledge did not differ significantly between native and non-native students. Natives mentioned 30 species, as well as non-natives, with frequency of citations of 153 plants ($\overline{x}=2.16;\mathrm{s}=1.92$) and 146 plants ($\overline{x}=1.86;\mathrm{s}=1.84$) in the free-listing, respectively. The most known and salient species were the same for both groups, with exceptions only for species mentioned by a single person and for *C. fluminensis* and *M. violaceus*, both only cited by two young natives. However, it is important to mention that 17 native participants (24.3% of the native participants) and 30 non-native participants did not mention any plants (37.4% of the non-native participants), although this difference was not significant (X^2^= 1.20; p = 0.27).

Analyzing only the domain of knowledge about native restinga plants, the same pattern is observed: young natives mentioned 21 species, with frequency of citations 134 plants ($\overline{x}=1.93;\mathrm{s}=1.90$) and young non-natives mentioned 19 species with frequency of citations 127 plants ($\overline{x}=1.60;\mathrm{s}=1.62$), revealing no significant differences in the knowledge of the two groups. Again, the difference in known species was only due *C. fluminensis* and *M. violaceus*.

### Which plants uses do the youth know?

Among all 37 species mentioned (natives and exotics), 24 species had a use indicated in the interviews and 13 did not. The participants listed 147 uses in all ($\overline{x}=1.87;\mathrm{s}=1.83$). The category with the highest frequency of citations was edible (93), followed by medicinal (32) and ornamental (14). Data on the distribution of knowledge about plant uses (see Additional file: Table 3) showed no significant difference between native and non-native students. The frequency of citations per category of uses (Tables 1 and 3) showed that the most cited categories were the same for native and non-native students: edible, medicinal, and ornamental.

When we analyzed only the domain of knowledge about native restinga species, the data showed that of the 24 native Brazilian restinga species mentioned, 16 had a use indicated by the students and 8 did not (Table 1). The distribution of knowledge about plant uses with relation to the students’ origin did not differentiate significantly (see Additional file: Table 3). The ten main useful native species in terms of citation frequency were *P. arrabidae* (76), *A. arenaria* (36), *E. uniflora* (33), *C. icaco* (22), *S. terebinthifolia* (15), *A. occidentale* (12), *P. caimito* (8), *C. guttata* (8), *D. ambrosioides* (6), and *M. Floribunda* (6), with the most represented families being Cactaceae (4), Arecaceae (3), and Myrtaceae (3). Only one or two species from other families were cited. It is important to mention that during the interviews, 89 youth (59.4% of the participants) reported that they did not currently use native restinga plants. Another important note is that the answers provided in the questionnaires did not detail or specify the procedures of using the plants.

### From whom do the youth learn?

The participants of this study mentioned 13 learning models, of which teacher (31), mother (24), and grandmother (20), followed by media (19), friends (17), father (12), and older people (11) were mentioned frequently. With a lower citation frequency, grandfather (5), scoutmaster (3), great-grandmother (2), aunt (2), uncle (1), and nutritionist (1) were also reported. Only one participant mentioned three models, and the other 103 who mentioned at least one plant recognized only one or two models. The main models for native and non-native youth were the same: teachers, with 16 and 15 citations, respectively, and mother, with 13 and 11 citations, respectively. Parents were the only model cited significantly more by young natives (9 citations) than non-natives (3 citations); all other models had close citation frequencies. Cultural transmission about restinga plants occurred mainly through family and school (vertical and oblique one-to-many cultural transmission modes, see Additional file: Table 4). The teacher was the most cited transmitter, followed by mother, and formal class and family interactions were the main ways of learning.

Frequencies of transmission modes differed among students (X^2^ = 52.26; df = 3; p < 0.001), with the vertical and one-to-many modes being the most important. The horizontal and many-to-one pathways did not differ (X^2^ = 0.29, df = 1, p = 0.59), and were less frequent in the studied population. This result partially corroborates our first hypothesis, that vertical transmission is the most important mode for research partners and adolescent students. However, the data indicated that one-to-many is also a fundamental mode for sharing information about restinga plants. The geographic origin of the student (native or non-native) was not associated with the form of knowledge transmission (vertical, horizontal, one-to-many, and many-to-one) (X^2^ = 0.74, p = 0.8637). This result refutes our second hypothesis, that the horizontal mode (diffusive) would be relatively more important to non-native students than the native ones.

### How do the youth learn?

Data from the questionnaires showed that the young learned through formal school classes (25), conversations (23), visiting restinga environments (21), family interactions (19), accessing media (19), and observations of medical and food preparation by others (6). Only five individual learning events were reported. When these citations are categorized into cognitive processes, it becomes apparent that teaching (formal and informal), with 90 citations, is the most important cultural transmission process (X^2^ = 138.41, df = 2, p < 0.001). Observation and individual learning, with only 7 and 5 citations, respectively, did not differ from each other (X^2^ = 0.33, df = 1, p = 0.56). Thus, we corroborate our third hypothesis that teaching is the most important mechanism for knowledge transmission.

### What is the context or stimulus for learning?

**Fig. 2:**
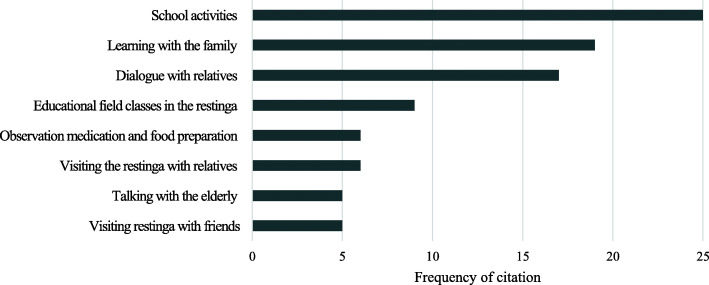
Contexts of learnings mentioned by the students of the schools EMFPA, EMFLS, and CMRB

The information in the questionnaires showed that the cultural transmission context (or stimuli) was mainly lectures and school activities (25 citations), direct learning with the family (19), dialog with relatives (17), educational field classes in the restinga (9), observation of medication and food preparation by family members (6), visiting the restinga with relatives (6), talking with the elderly (5), and visiting the restinga for leisure with friends (5) (Fig. 2). However, there is no evidence that any of these processes is statistically more important than another (X^2^ = 1.8, df = 3, p = 0.61).

## Discussion

The results showed that there is no strong evidence that the knowledge about restinga plants varies in relation to the origin of young students and also did not point out differences between the ways of acquiring it. For both groups, native and non-native, the most prominent plants and transmission modes (vertical and oblique one-to-many) were the same. This may mean that the non-native families are already well established in the region and know the plants well, passing this knowledge on to their children. In addition, the prominence of the teacher may indicate that subjects related to restinga are being taught in schools, even though our investigation was limited to students. In this sense, other studies involving other actors, such as teachers, have indicated that the contextualization of formal schooling with local knowledge has strengthened the latter and contributed to its perpetuation [50].

The results revealed that schools and teachers tend to contribute significantly to the process of transmission and the perpetuation of local ecological knowledge, especially in recent urbanized areas such as the one studied. Investigations carried out in rural and indigenous communities have shown that non-contextualized schooling negatively affects traditional knowledge about plants and that curriculum content is not relevant to the daily lives of young people [10, 26–28]. Another study [51], carried out in the Brazilian semi-arid region, on students’ knowledge about wild vertebrates pointed out that school was one of the ways of accessing knowledge, however, not the main one. The present study, carried out in urban areas in which traditional communities have been losing space and young people are already inserted in a post-industrial scenario, the professor acted as an important transmitter about local plants, as far as we know, even in the absence of an official program of contextualization. This may be a reflection of punctual actions by some teachers in their contextualize classes. However, it is important to emphasize that the official recommendations of the Ministry of Education of Brazil, recognizing the high Brazilian socio-cultural diversity, propose that students develop the ability to recognize the interactions between different societies and nature [52]. Therefore, the engagement and valorization of traditional ethnobotanical knowledge should be encouraged in local curricula.

The most of the informants know the resources of the surroundings, especially edible and medicinal plants common in the restinga (*P. arrabidae*, *A. arenaria*, *E. uniflora*, *C. icaco*, *S. terebinthifolia*, and *A. occidentale*), corroborating studies in the Cabo Frio region [43] and following the trend found in several studies that the childhood is the most important phase for learning in different social groups [1, 3, 8, 18, 19, 53–56]. However, almost a third of the interviewees did not know about restinga plants. This may indicate a distancing of young people with natural resources and time spent in nature, reducing connecting with plants, as is observed in areas undergoing urbanization and livelihoods changelings [27, 28, 57, 58], which may be occurring in the Cabo Frio region, recently urbanized. In this sense, the restinga plants are low present in the daily activities of those students. In addition, the study region has been experiencing a great increase in violence and crime. According to official Brazilian statistics [59], Cabo Frio is among the 20 most dangerous cities in the Rio de Janeiro state, with 37% homicides increase in 2020. The insecurity has led to a decrease in visits by local populations to the restinga environment which are isolated places with no policing and dangerous in local perception.

In situations where there is a strong dependence on natural resources, children achieve competence in certain cognitive domains at an early age [1, 7, 9, 10, 53–56], usually before the age of 12. For example, among the Aka pygmy people, 10-year-old boys are able to collect resources and prepare food, and they possess special skills that will not change as teenagers or adults [1]. However, in the studied reality, it is believed that complete sufficiency is lacking. After all, despite having local knowledge about restinga plants, the students cited only two species on average, which was not representative knowledge in relation to the ethnobotanical studies conducted with adults in the same region by Fonseca-Kruel and Peixoto [43]. These authors carried out an ethnobotanical survey of the restinga species used by traditional fishermen (adults). But in the last five decades the traditional activities have been declining due to new opportunities provided by industrialization. Similar results were found by Ruiz-Mallen et al. [50] with adolescent students in a community undergoing urbanization in México. In this sense, possibly the knowledge and use of wild plants is no longer as present in people’s daily lives as it was in the past, a reflection of local changes in lifestyle and interest, provided by new post-industrial facilities. This could be related to the smaller number of plants known to the younger generation, which are in an urban setting. Also, this result indicates that knowledge about native vegetation is present in a small circle of people and not with general public. However, to clarify this hypothesis, investigations with local specialties and their children are required.

Socioeconomic factors can influence the construction of local knowledge. For example, several studies indicate that urbanization may reduce local knowledge, when compared to rural realities, due to less dependence on the environment and greater access to markets and services [21, 60, 61]. Despite the negative trend between urbanization and level of contact with wild plants, an interesting finding is that the local names provided by the young people were at the species level, despite the low average number of plants listed. This can reinforce the importance of these species in the local knowledge system, especially when analyzing the categories of use. A study by Eyssartier et al. [58] in urban, peri-urban, and rural schools showed that students of the latter had greater knowledge about plants, especially in the edible, medicinal, and ornamental domains, the same domains highlighted in this study. Our data suggest adaptive knowledge, since native plants used for food were the best known, and the medicinal category was the second most important. The human mind tends to favor adaptive information [16, 62], it is to be expected that the information of initial learning would be linked to social memories related to survival [6].

A deeper look at the vertical and oblique one-to-many modes showed in our results, highlighting the role of mothers and/or grandparents as learning models, which is common in South America, where women are often responsible for the education of offspring [18, 19, 27]. In the early stages of life, the vertical path is the most important in the transmission of knowledge (for example, [1, 20, 56, 63–66]). After all, it is expected that those actors who occupy most of the space and time of childhood, parents, are more important for transmission [65]. In the communities of Cabo Frio region, the women were responsible for family nutritional and healthcare [67], reinforcing their role in sharing knowledge about edible and medicinal plants. Mothers and grandparents, who are often in charge of nutritional and medicinal responsibilities, are usually trusted by children, representing secure sources of information for them as noted in other studies conducted in South America [19, 27, 68–70].

Although records indicate that parents are the main role models for infants, the pattern can change with the young. The data from the investigated reality suggest that students are starting a phase in which pathways besides their parents are important to them (oblique one-to-many). A similar reality was recorded by Mathez-Stiefell and Vandebroek [71] that, for two Andean groups, the vertical and horizontal modes were equally important in the perception of the informants. In our case, the new models are represented by teachers, possibly because they are people with high social recognition.

With development, people access new models available for copying information [1, 3, 10, 29, 65], like teachers and formal schooling. Therefore, non-parental models become more important in learning at older ages; in other words, the pattern of transmission may change throughout life [10, 29]. For example, Haselmair et al. [70] and Reyes-García et al. [29] pointed out that when children grow up, they begin interacting with actors outside the family nucleus, which results in non-conservative pathways, such as the horizontal, becoming more important in learning. However, individuals of the same age can only pass on elementary knowledge and skills [29], which may explain the low representativeness of the horizontal mode in studied reality. Therefore, accessing information about restinga plants would require specialists as models, like teachers and experts (oblique one-to-many), which is in agreement with studies that emphasize that prestige is one of the biases that that guides social learning [15, 72].

It is interesting to note that the learning pattern did not vary with the origin of the informant. We hypothesized that non-native youth, as they arrive in a new and unknown environment, would tend to learn from local peers, in the case, the horizontal mode being more prominent from them than the vertical mode. However, this did not happen. Vertical transmission was as important for non-natives as it was for natives, and the same pattern was observed for the oblique one-to-many mode. This results may suggest that the immigrant families are incorporating and sharing some dimensions of local knowledge about restinga plants, as seen in southern Brazil [24] and that regardless the youth origin, prestigious is a strong force in choosing models to learn from [15, 16, 18].

The classic models of transmission [1, 63] state that vertical transmission results in a highly heterogeneous cultural system with very slow evolution, as innovations are restricted to family core. Diffusive pathways, like one-to-many, otherwise contribute to more homogeneous knowledge systems and high improvement rates. In the investigated reality, these two pathways with completely different implications were influential. Therefore, what is the relationship between the social learning structure and the distribution and evolution of local knowledge? The result is a system rich in idiosyncrasies, and therefore quite heterogeneous, as shown by many species being cited by one or two people. Only six species were cited by more than ten informants. Heterogeneity is believed to be the result of parental learning. However, in such a heterogeneous system, what would be the contribution of one-to-many learning events? We see two explanations for this. First, because the recognition of teachers as models to learn about local ecological knowledge may be recent in the lives of informants who, until then, learned from their parents, these new pathways may not have yet resulted in the distribution of information. Second, the presence of teachers as an important mode can be a methodological device, as the results were obtained only in schools, which means the data can be biased.

In the investigated reality, teaching (informal and formal) was the most important process of transmitting knowledge, although there are several other/s, such as observation and imitation (see [73]). From an evolutionary psychology perspective, teaching is a cognitive adaptation exclusive to human beings, which allows the most reliable transmission of very complex or subjective knowledge and skills [74]. In addition, from an adaptive perspective, teaching can be a more efficient cognitive process than observation because it is more flexible; that is, it does not require the realization of a skill or knowledge to be transmitted. For example, Soldati et al. [5], who studied groups of farmers, found that mothers explicitly teach their children about medicinal plants, even without the manifestation of a certain disease.

## Conclusions

The distribution and transmission of knowledge about useful restinga plants did not vary based on the socioeconomic factor origin, showing that immigration events and insertion in a new environment have not changed the processes of social learning. The plants known best are those for edible and medicinal use, which is evidence of adaptive knowledge linked to material survival demands. The vertical and one-to-many modes are the most important, indicating that throughout the development of youth, leaving childhood; models besides family members become significant, with emphasis on prestige individuals (teachers). Direct teaching was important as a transmission process during youth, with family and with formal education, showing the importance of this cognitive process in cultural evolution. Regarding learning strategies, they were defined by the nature of the information (relevant for survival), in the case, food and medicine, and not to the socio-environment context, since immigrants learned the same cultural information with the same models and process, even when facing a new environment. In this sense, even that the knowledge about restinga plants is not extensive among the participant students (regardless of being native or non-native), the young from immigrant families are incorporating local plant knowledge. We pointed out that this study follows global trends of changes in cultural transmission, possibly due to human pressures such as the extensive loss of vegetation cover and livelihood changes due to urbanization, in which the reduction of the young people’s connection with nature may be related to the reduced knowledge about the local flora. To help revise this situation, the school and the teacher showed to be a new opportunity and strategy for transmitting traditional/local knowledge, which can also contribute to the biocultural conservation of restinga species. Finally, our findings indicate that formal education can be used in urban communities to engage traditional knowledge.

## Supplementary Information

**Additional file 1: Table 2**. Distribution of plant knowledge in relation to the young people origin and geographic origin of plant species (exotic and native from Brazilian restinga vegetation). **Table 3**. Distribution of useful restinga plant knowledge in relation to the young people origin. **Table 4**. Absolute frequencies and relative frequencies (FR%) of cultural transmission modes according to the origin of the young of the schools EMFPA, EMFLS and CMRB in Cabo Frio Region.

## Data Availability

The datasets used and/or analyzed during the current study are available from the corresponding author on reasonable request. Part of data analyzed during this study is included in supplementary information files in this published article.

## Abbreviations

EMFPA: Escola Municipal Francisco Porto Aguiar
EMFLS: Escola Municipal Francisco Luiz Sobrinho
CMRB: Colégio Municipal Rui Barbosa
CSCG: Center for Scientific Computing and Geoprocessing
JBRJ: Instituto de Pesquisas Jardim Botânico do Rio de Janeiro
