# Supplementary material for: The role of schools as an opportunity for transmission of local knowledge about useful Restinga plants: experiences in southeastern Brazil
Source: J Ethnobiol Ethnomed. 2021 May 17;17:34. doi: 10.1186/s13002-021-00461-0 (PMC8130333; doi:10.1186/s13002-021-00461-0)
Supplement: Supplementary file 1 — Additional file 1: Table 2. Distribution of plant knowledge in relation to the young people origin and geographic origin of plant species (exotic and native from Brazilian restinga vegetation). Table 3. Distribution of useful restinga plant knowledge in relation to the young people origin. Table 4. Absolute frequencies and relative frequencies (FR%) of cultural transmission modes according to the origin of the young of the schools EMFPA, EMFLS and CMRB in Cabo Frio Region. [file 13002_2021_461_MOESM1_ESM.docx]

**Additional file**

**Table 2.** Distribution of plant knowledge in relation to the young people origin (natives and non-natives) and geographic origin of plant species (exotic and native from Brazilian restinga vegetation).

|  | **Natives (N=70)** | **Non-natives (N=80)** | Total (N=150) |
| --- | --- | --- | --- |
| **Frequency of citation of all plants**  **(exotic and native)** | 153 | 146 | 299 |
| Mean and standard deviation | ($\overline{x}$=2.16; s = 1.92) | ($\overline{x}$=1.86; s = 1.84) | ($\overline{x}$=1.75; s = 1.73) |
| Number of species | 30 | 30 | 37 |
| **Frequency of citation of native restinga plants** | 134 | 127 | 261 |
| Mean and standard deviation | ($\overline{x}$=1.93; s = 1.90 ) | ($\overline{x}$=1.60; s = 1.62) | ($\overline{x}$=1.75; s = 1,73) |
| Number of restinga species (native) | 21 | 19 | 24 |
| **Frequency of citation of exotic plants** | 17 | 21 | 38 |
| Mean and standard deviation | ($\overline{x}$=0.24; s = 0.53) | ($\overline{x}$=0.26; s =0.63) | ($\overline{x}$=0.25.; s =0.59) |
| Number of exotic species | 9 | 11 | 13 |

**Table 3.** Distribution of useful restinga plant knowledge in relation to the young people origin.

| **Young people origin** | **Natives (N=70)** | **Non-natives (N=80)** | **Total (N=150)** |
| --- | --- | --- | --- |
| **Number of useful species (exotic and native)** | 18 | 18 | 24 |
| *Frequency of citation of uses*  *(all species – exotic and native)* | *78* | *69* | *147* |
| *Mean and standard deviation* | *(*$\overline{x}$*=2.07; s = 1.83)* | *(*$\overline{x}$*= 1.70; s = 1.75)* | *(*$\overline{x}$*=1.87; s=1.83)* |
| **Number of restinga useful species (natives)** | 12 | 13 | 16 |
| *Frequency of citation of*  *restinga native plants uses* | *69* | *60* | *129* |
| *Mean and standard deviation* | *(*$\overline{x}$*=0.94; s = 1.34)* | *(*$\overline{x}$*=0.79; s = 1.17)* | *(*$\overline{x}$*=0.86; s = 0.35)* |
| **Frequency of citation per category of use of native restinga plants** | | | |
| Edible | 46 | 41 | 87 |
| Medicinal | 12 | 14 | 26 |
| Ornamental | 7 | 5 | 12 |
| Tinctorial | 2 | 0 | 2 |
| Handicraft | 1 | 0 | 1 |
| Construction | 1 | 0 | 1 |

**Table 4.** Absolute frequencies and relative frequencies (FR%) of cultural transmission modes (vertical, horizontal, one-to-many and many-to-one) according to the origin (native/non-native) of the young collaborators of the schools EMFPA, EMFLS and CMRB in Cabo Frio Region.

|  | Vertical | One-to-many | Horizontal | Many-to-one | Sum | FR Vertical  (%) | FR One-to-many (%) | FR Horizontal (%) | FR Many-to-one (%) |
| --- | --- | --- | --- | --- | --- | --- | --- | --- | --- |
| **Native** | 35 | 27 | 9 | 6 | 77 | 45,454 | 35,064 | 11,688 | 7,7920 |
| **Non-native** | 29 | 27 | 8 | 8 | 72 | 40,277 | 37,500 | 11,111 | 11,111 |
| *Total* | 64 | 54 | 17 | 14 | 149 |  |  |  |  |
